# Association between Food Group Intake and Serum Total Cholesterol in the Japanese Population: NIPPON DATA 80/90

**DOI:** 10.2188/jea.JE20090227

**Published:** 2010-05-05

**Authors:** Imako Kondo, Kaori Funahashi, Mieko Nakamura, Toshiyuki Ojima, Katsushi Yoshita, Yosikazu Nakamura

**Affiliations:** 1 Faculty of Health Promotional Sciences, Hamamatsu University, Hamamatsu, Shizuoka, Japan; 2 Department of Community Health and Preventive Medicine, Hamamatsu University School of Medicine, Hamamatsu, Shizuoka, Japan; 3 School of Health Sciences, Fujita Health University, Toyoake, Aichi, Japan; 4 Nutritional Epidemiology Program, National Institute of Health and Nutrition, Tokyo, Japan; 5 Department of Public Health, Jichi Medical University, Shimotsuke, Tochigi, Japan

**Keywords:** serum cholesterol, Japan, nutrition, food, diet, survey

## Abstract

**Background:**

Dietary habit is one of the important determinants of health. Investigation of the association between diet and blood lipids at the food product level is more advantageous than that at the nutrient level because the results can be applied more directly to improving dietary habits for disease prevention.

**Methods:**

The integrated datasets of the NIPPON DATA and National Nutrition Surveys in Japan conducted in 1980 and 1990 were used for analysis. The association between serum total cholesterol concentration and food group intake was examined by multiple linear regression analysis separately for sex and survey year with data adjusted for age, body mass index and total energy intake.

**Results:**

Intakes of rice, sugar, sweets and snacks, fruit and vegetables other than green and yellow ones were lower and intakes of green and yellow vegetables, mushrooms, seaweed, eggs and milk were higher in the 1990 survey than in the 1980 survey. Intakes of meat, milk and eggs showed a positive association with serum total cholesterol concentration in both sexes while intake of legumes showed a negative association only in men in both the 1980 and 1990 surveys.

**Conclusions:**

Sex- and age-specific food group intakes for 1980 and 1990 were identified, and positive and negative associations between serum total cholesterol concentration and food group intake were revealed in a representative sample of the Japanese population. The results provide some insights into the improvements in dietary habits that can be made for disease prevention in Japan.

## INTRODUCTION

The prevention of arteriosclerotic disease has become increasingly important in Japan in recent times. The onset of arteriosclerotic disease is closely associated with risk factors such as blood lipids, blood pressure and several lifestyle factors, and prevention of the disease through lifestyle modification has been reported mainly in Western countries.^1^^,^^2^ Improving lifestyle through, for example, eating and exercise, has been recommended for the proper control of blood cholesterol concentration. Reducing intakes of meat, milk, dairy products and egg yolk while increasing intakes of vegetables and fruit has been shown to be effective for the prevention of coronary disease.^1^ Furthermore, the recommended amount of intake has been specified for each food group, nutrients such as fat and dietary fiber, and even detailed nutrients such as fatty acids.^2^ Studies at the nutrient or fatty acid level are important to clarify the effects of nutrient intake on blood lipid level, while studies at the food or food group level are of value in that the results can be applied more directly to the improvement of daily dietary habits. The present study aimed to reveal food group intake by sex and age group from data collected in 1980 and 1990, and to determine any association between serum total cholesterol concentration and food group intake in the Japanese general population.

## METHODS

The integrated datasets of NIPPON DATA80/90 (National Integrated Project for Prospective Observation of Non-communicable Disease And its Trends in the Aged) were used for data analysis. These datasets contained the results of the third (1980)^3^ and fourth (1990)^4^ National Surveys on Circulatory Disorders as baseline data, with additional data on nutritional intake per individual estimated from the results of the National Nutrition Surveys in Japan^5^^,^^6^; hereinafter referred to as the 1980 survey and the 1990 survey. The details of the datasets have been reported elsewhere.^7^ In the present study, data from subjects who consumed 500–5000 kcal/day of total energy were included for analysis; seven subjects from the 1980 survey and one subject from the 1990 survey whose total energy intake was less than 500 kcal/day, and eight subjects from the 1980 survey and one subject from the 1990 survey whose total energy intake was more than 5000 kcal/day were excluded.

For data analysis, we used 17 food groups from among the 18 food groups and 2 subgroups (rice and wheat from cereals) defined in the National Nutrition Survey in Japan: rice, wheat, nuts and seeds, potatoes, sugar and preserves (hereafter sugar), sweets and snacks, fat and oil, legumes, fruit, green and yellow vegetables, other vegetables (ie, vegetables other than green and yellow ones), mushrooms, seaweed, fish and shellfish (hereafter fish), meat and poultry (hereafter meat), eggs, and milk and dairy products (hereafter milk). Cereals were not used for the analysis because they are mainly composed of rice and wheat, and intake of cereals other than rice and wheat (eg, *soba* and cornflakes) were heterogeneous and rather small. Moreover, we did not use the food groups “seasonings and beverages” and “other food” because both of these food groups were heterogeneous and their nutritional significance was thus considered difficult to interpret.

Statistical analysis involved the following. The means and standard deviations of intake of each food group by sex and age group for each survey year were calculated. Multiple linear regression analysis was then performed separately for sex and survey year with serum total cholesterol concentration (mg/dl) as the objective variable and age (years), body mass index (kg/m^2^), total energy intake (kcal/day) and intake of food groups (g/day) as the explanatory variables (covariates). Data for the 17 food group intakes were entered into the model first separately (model 1) and then collectively (model 2). The intake of legumes, which was significantly negatively correlated with serum total cholesterol concentration only in men, was also analyzed in a population of women aged 55 years or older. Differences were considered significant at *P* < 0.05. All statistical analysis was performed using SPSS® 17.0J.

## RESULTS

Mean food group intakes were analyzed for 4578 men and 5829 women in the 1980 survey and 3487 men and 4853 women in the 1990 survey (Tables 1 and 2). Intakes of rice, sugar, sweets and snacks, fruit and other vegetables were lower and intakes of green and yellow vegetables, mushrooms, seaweed, eggs and milk were higher in the 1990 survey than in the 1980 survey, in both men and women. Because the classification of green and yellow vegetables was somewhat different between the two surveys,^5^^,^^6^ the combined intakes of green and yellow vegetables and other vegetables (ie, total vegetables) was examined and found to be similar in both surveys.

**Table 1.** Mean food group intake in men by survey year (g/day)

Survey year	Food group	Age (year)											

		30–39		40–49		50–59		60–69		70+		Total	
					
		Mean	(SD)	Mean	(SD)	Mean	(SD)	Mean	(SD)	Mean	(SD)	Mean	(SD)
		*n* = 1217		*n* = 1196		*n* = 1018		*n* = 676		*n* = 471		*n* = 4578	
1980	Rice	285.2	(94.1)	307.8	(99.3)	331.6	(102.6)	305.3	(109.5)	264.0	(88.7)	302.2	(101.4)
	Wheat	110.8	(62.3)	90.8	(52.6)	68.3	(48.9)	73.6	(53.6)	63.2	(53.4)	85.7	(57.6)
	Nuts and Seeds	1.2	(3.9)	1.4	(5.0)	1.7	(6.0)	1.1	(3.2)	1.6	(4.6)	1.4	(4.7)
	Potatoes	62.0	(43.4)	63.4	(40.8)	70.4	(49.9)	71.4	(54.8)	67.4	(50.6)	66.2	(47.0)
	Sugar and Preserves	13.5	(9.4)	12.9	(9.5)	15.2	(12.2)	14.0	(10.7)	13.0	(10.0)	13.7	(10.4)
	Sweets and Snacks	15.2	(15.2)	14.0	(14.9)	11.7	(14.1)	18.4	(19.8)	23.9	(28.7)	15.5	(17.8)
	Fat and Oil	22.1	(13.3)	18.5	(11.4)	16.9	(10.8)	13.1	(9.8)	9.3	(8.5)	17.4	(12.0)
	Legumes	70.6	(39.8)	77.7	(45.3)	93.1	(54.3)	94.0	(54.8)	82.6	(51.9)	82.2	(49.2)
	Fruit	106.9	(72.3)	142.4	(89.3)	150.6	(103.2)	168.0	(105.7)	169.0	(122.6)	141.3	(97.7)
	Green and Yellow ​ Vegetables	51.9	(35.3)	57.6	(37.6)	62.0	(41.8)	58.2	(44.0)	52.1	(40.2)	56.6	(39.4)
	Other Vegetables	217.8	(87.2)	229.7	(92.9)	245.6	(111.4)	247.5	(108.4)	202.6	(93.8)	229.9	(99.4)
	​ [Total Vegetables]	269.8	(101.0)	287.2	(106.4)	307.6	(124.1)	305.6	(122.4)	254.6	(107.4)	286.5	(113.2)
	Mushrooms	9.3	(11.8)	9.9	(13.6)	11.6	(14.5)	10.5	(13.8)	7.8	(11.5)	10.0	(13.2)
	Seaweed	5.5	(7.6)	6.5	(8.2)	7.3	(8.7)	6.8	(9.1)	6.1	(7.6)	6.4	(8.3)
	Fish and Shellfish	113.7	(58.4)	128.3	(65.5)	142.4	(69.8)	125.4	(60.2)	101.1	(53.3)	124.3	(64.0)
	Meat and Poultry	87.4	(43.2)	78.7	(41.0)	68.2	(38.9)	52.8	(31.8)	41.3	(29.7)	71.0	(41.7)
	Eggs	44.5	(22.6)	42.2	(21.9)	42.2	(22.4)	35.3	(21.8)	31.5	(19.9)	40.7	(22.4)
	Milk and Dairy ​ Products	74.4	(52.0)	68.1	(49.4)	69.4	(63.7)	76.3	(68.8)	73.6	(70.6)	71.8	(58.9)

		*n* = 659		*n* = 836		*n* = 793		*n* = 708		*n* = 491		*n* = 3487	
1990	Rice	250.0	(72.9)	261.8	(79.2)	282.6	(88.6)	255.9	(85.6)	224.7	(76.0)	257.9	(83.0)
	Wheat	100.3	(53.9)	86.3	(49.9)	72.1	(51.9)	76.2	(55.0)	68.8	(50.6)	81.2	(53.4)
	Nuts and Seeds	1.1	(3.1)	1.3	(3.7)	2.0	(5.6)	1.8	(4.5)	1.8	(4.6)	1.6	(4.4)
	Potatoes	62.4	(40.6)	62.3	(36.7)	72.8	(48.2)	72.7	(47.3)	71.2	(47.8)	68.1	(44.3)
	Sugar and Preserves	11.1	(9.5)	12.2	(8.9)	13.0	(10.8)	12.6	(9.0)	12.2	(10.0)	12.2	(9.6)
	Sweets and Snacks	13.0	(14.8)	12.7	(14.3)	9.9	(14.2)	15.9	(22.1)	19.3	(24.1)	13.7	(18.0)
	Fat and Oil	22.9	(11.1)	19.5	(10.3)	17.8	(9.8)	15.1	(9.6)	10.6	(7.6)	17.6	(10.6)
	Legumes	71.0	(42.4)	78.5	(46.1)	96.7	(53.3)	95.5	(53.4)	88.1	(53.7)	86.0	(50.8)
	Fruit	76.8	(58.5)	107.6	(75.8)	131.0	(93.2)	146.1	(103.7)	161.9	(110.6)	122.6	(93.2)
	Green and Yellow ​ Vegetables	76.6	(42.1)	79.2	(44.1)	95.7	(56.4)	95.5	(60.7)	84.2	(52.0)	86.5	(52.1)
	Other Vegetables	180.4	(79.3)	189.2	(78.2)	204.8	(88.8)	201.4	(87.3)	172.8	(83.5)	191.2	(84.3)
	​ [Total Vegetables]	257.0	(99.3)	268.4	(98.2)	300.5	(115.0)	296.8	(114.1)	257.0	(105.1)	277.7	(108.3)
	Mushrooms	10.1	(12.0)	12.1	(12.8)	15.6	(18.9)	12.9	(15.5)	10.8	(13.6)	12.5	(15.0)
	Seaweed	6.0	(6.7)	7.2	(8.2)	8.8	(10.3)	8.3	(12.3)	7.2	(8.8)	7.6	(9.5)
	Fish and Shellfish	107.6	(50.5)	127.7	(56.0)	148.9	(69.1)	128.3	(56.6)	109.4	(53.4)	126.3	(59.9)
	Meat and Poultry	88.4	(41.4)	86.1	(37.8)	70.5	(39.1)	55.8	(32.5)	43.5	(28.4)	70.8	(40.1)
	Eggs	47.2	(23.5)	47.1	(22.1)	47.8	(23.9)	42.4	(22.8)	39.7	(23.5)	45.3	(23.3)
	Milk and Dairy ​ Products	85.8	(58.6)	79.5	(61.7)	90.9	(79.0)	99.0	(83.9)	109.9	(90.8)	91.5	(75.3)

**Table 2.** Mean food group intake in women by survey year (g/day)

Survey year	Food group	Age (year)											

		30–39		40–49		50–59		60–69		70+		Total	
					
		Mean	(SD)	Mean	(SD)	Mean	(SD)	Mean	(SD)	Mean	(SD)	Mean	(SD)
		*n* = 1583		*n* = 1467		*n* = 1316		*n* = 897		*n* = 566		*n* = 5829	
1980	Rice	185.1	(62.2)	212.7	(71.3)	216.9	(75.0)	225.8	(74.6)	223.4	(76.1)	209.2	(72.5)
	Wheat	113.2	(52.9)	83.3	(51.9)	73.6	(52.0)	64.6	(45.2)	49.6	(37.9)	83.1	(54.1)
	Nuts and Seeds	1.4	(5.1)	1.4	(4.7)	2.3	(7.2)	1.8	(5.7)	1.7	(4.8)	1.7	(5.6)
	Potatoes	57.6	(35.7)	62.9	(42.7)	67.2	(50.2)	71.7	(51.9)	67.5	(54.0)	64.2	(45.8)
	Sugar and Preserves	13.4	(8.9)	13.3	(10.1)	14.0	(10.9)	12.2	(9.8)	11.8	(9.1)	13.2	(9.9)
	Sweets and Snacks	28.6	(27.0)	29.3	(32.1)	28.3	(32.7)	21.7	(24.8)	19.8	(24.6)	26.8	(29.4)
	Fat and Oil	19.0	(11.0)	17.8	(10.7)	14.6	(9.7)	11.5	(8.9)	9.5	(7.1)	15.6	(10.5)
	Legumes	57.6	(32.4)	70.4	(40.1)	82.9	(48.0)	77.5	(46.9)	72.7	(43.7)	71.1	(42.7)
	Fruit	147.1	(88.2)	195.1	(121.9)	219.7	(143.1)	205.0	(139.7)	168.4	(120.7)	186.5	(125.2)
	Green and Yellow ​ Vegetables	51.4	(32.3)	60.0	(39.8)	63.8	(45.4)	60.4	(45.0)	58.8	(46.1)	58.5	(41.1)
	Other Vegetables	191.9	(76.7)	218.7	(92.4)	227.9	(98.3)	213.4	(95.1)	187.7	(91.4)	209.7	(91.4)
	​ [Total Vegetables]	243.3	(88.0)	278.7	(108.2)	291.4	(113.7)	273.7	(110.2)	246.4	(108.4)	268.1	(106.5)
	Mushrooms	7.9	(9.8)	9.6	(12.0)	10.3	(13.2)	9.0	(12.6)	7.5	(10.7)	9.0	(11.7)
	Seaweed	4.3	(6.0)	6.4	(7.9)	7.2	(9.4)	6.9	(8.8)	6.4	(7.0)	6.1	(7.9)
	Fish and Shellfish	86.6	(42.1)	98.2	(50.6)	106.0	(50.6)	98.2	(49.4)	89.7	(47.1)	96.0	(48.4)
	Meat and Poultry	64.1	(34.3)	64.0	(33.5)	51.1	(33.8)	39.3	(27.1)	32.3	(25.5)	54.2	(34.2)
	Eggs	38.5	(19.2)	36.9	(18.9)	34.7	(19.9)	31.7	(21.1)	26.6	(16.4)	35.0	(19.7)
	Milk and Dairy ​ Products	102.7	(61.9)	90.7	(72.0)	92.7	(83.4)	82.9	(73.1)	68.9	(62.0)	91.1	(72.1)

		*n* = 1031		*n* = 1171		*n* = 1034		*n* = 915		*n* = 702		*n* = 4853	
1990	Rice	163.1	(48.9)	178.9	(52.5)	185.7	(60.6)	188.6	(66.4)	187.2	(62.4)	180.0	(58.6)
	Wheat	96.0	(46.6)	82.7	(45.2)	72.5	(49.2)	66.0	(46.6)	55.3	(42.8)	76.2	(48.2)
	Nuts and Seeds	1.2	(3.0)	1.6	(4.5)	1.9	(5.4)	1.8	(4.2)	1.6	(4.5)	1.6	(4.4)
	Potatoes	58.1	(33.3)	63.0	(41.1)	68.2	(44.9)	75.2	(50.0)	67.0	(45.6)	65.9	(43.3)
	Sugar and Preserves	10.9	(7.2)	12.3	(9.7)	11.9	(9.0)	11.8	(10.5)	11.5	(9.2)	11.7	(9.2)
	Sweets and Snacks	23.7	(23.2)	26.2	(30.2)	23.3	(32.3)	20.1	(26.3)	18.5	(24.2)	22.8	(27.9)
	Fat and Oil	20.0	(9.5)	18.9	(9.6)	15.2	(8.8)	13.1	(8.6)	10.1	(6.7)	16.0	(9.5)
	Legumes	58.0	(30.8)	69.5	(40.7)	82.1	(45.0)	82.9	(48.9)	73.2	(42.3)	72.8	(42.7)
	Fruit	105.8	(76.6)	152.3	(104.1)	187.6	(130.4)	184.3	(120.3)	149.4	(114.5)	155.6	(114.0)
	Green and Yellow ​ Vegetables	73.0	(40.3)	84.8	(50.5)	98.6	(60.8)	95.2	(58.7)	83.4	(53.6)	87.0	(53.8)
	Other Vegetables	159.3	(61.6)	180.2	(74.4)	190.0	(85.9)	179.0	(84.5)	154.7	(73.3)	174.0	(77.5)
	​ [Total Vegetables]	232.3	(80.1)	264.9	(99.6)	288.6	(115.0)	274.3	(109.1)	238.2	(97.6)	260.9	(103.2)
	Mushrooms	9.5	(10.5)	12.6	(13.5)	14.1	(16.5)	11.6	(14.1)	8.6	(10.9)	11.5	(13.6)
	Seaweed	4.9	(5.4)	7.2	(8.3)	8.5	(10.4)	8.1	(10.8)	7.5	(9.4)	7.2	(9.1)
	Fish and Shellfish	82.6	(36.8)	102.4	(43.2)	110.7	(49.4)	98.0	(44.1)	90.3	(43.1)	97.4	(44.6)
	Meat and Poultry	66.0	(31.0)	68.0	(30.9)	53.0	(30.1)	43.1	(26.1)	34.6	(23.8)	54.8	(31.5)
	Eggs	42.8	(20.0)	41.1	(19.5)	39.8	(20.2)	35.5	(20.2)	33.1	(19.1)	39.0	(20.1)
	Milk and Dairy ​ Products	114.4	(71.8)	110.2	(84.7)	113.8	(96.9)	111.9	(90.3)	99.1	(89.2)	110.6	(86.8)

The association between serum total cholesterol concentration and food group intake was analyzed for those with available data (4569 men and 5818 women in the 1980 survey, 3220 men and 4494 women in the 1990 survey) (Table 3). The means and standard deviations of serum total cholesterol concentration and body mass index were 186.4 ± 32.8 mg/dl and 22.5 ± 2.9 kg/m^2^ in men and 191.2 ± 34.0 mg/dl and 22.8 ± 3.4 kg/m^2^ in women in the 1980 survey, and 198.6 ± 36.8 mg/dl and 22.9 ± 3.0 kg/m^2^ in men and 206.9 ± 38.8 mg/dl and 22.8 ± 3.3 kg/m^2^ in women in the 1990 survey, respectively.

**Table 3.** Associations between food group intake and total cholesterol level using multiple regression analysis

Survey year	Food group	Men				Women			
	
		model 1^a^		model 2^b^		model 1^a^		model 2^b^	
			
		β^c^	*P*	β^c^	*P*	β^c^	*P*	β^c^	*P*
1980	Rice	−0.155	<0.001	−0.096	0.004	−0.149	<0.001	−0.089	0.003
	Wheat	0.063	<0.001	−0.001	0.970	0.058	<0.001	−0.011	0.513
	Nuts and Seeds	0.000	0.978	−0.010	0.500	0.020	0.107	0.006	0.612
	Potatoes	−0.036	0.014	−0.033	0.033	−0.064	<0.001	−0.064	<0.001
	Sugar and Preserves	0.022	0.140	0.000	0.996	0.054	<0.001	0.037	0.006
	Sweets and Snacks	0.019	0.201	−0.008	0.654	0.019	0.147	−0.008	0.595
	Fat and Oil	0.087	<0.001	0.021	0.279	0.084	<0.001	0.016	0.348
	Legumes	−0.047	0.002	−0.036	0.023	−0.021	0.102	−0.012	0.379
	Fruit	0.017	0.273	−0.013	0.433	0.033	0.014	−0.001	0.962
	Green and Yellow Vegetables	0.027	0.068	0.016	0.289	0.035	0.006	0.018	0.173
	Other Vegetables	−0.040	0.009	−0.035	0.028	−0.028	0.036	−0.026	0.068
	Mushrooms	−0.011	0.438	−0.009	0.554	0.007	0.595	0.007	0.564
	Seaweed	−0.032	0.025	−0.022	0.125	−0.020	0.105	−0.012	0.358
	Fish and Shellfish	0.004	0.791	0.030	0.073	−0.026	0.044	−0.010	0.496
	Meat and Poultry	0.109	<0.001	0.071	<0.001	0.085	<0.001	0.044	0.009
	Eggs	0.071	<0.001	0.036	0.019	0.066	<0.001	0.032	0.015
	Milk and Dairy Products	0.105	<0.001	0.068	<0.001	0.118	<0.001	0.083	<0.001

1990	Rice	−0.093	<0.001	−0.036	0.255	−0.122	<0.001	−0.089	0.001
	Wheat	0.062	<0.001	0.030	0.177	0.042	0.004	0.001	0.941
	Nuts and Seeds	0.038	0.027	0.037	0.036	0.020	0.149	0.008	0.598
	Potatoes	−0.070	<0.001	−0.053	0.004	−0.006	0.684	−0.006	0.677
	Sugar and Preserves	0.021	0.235	0.017	0.353	0.029	0.051	0.021	0.165
	Sweets and Snacks	−0.050	0.005	−0.055	0.005	−0.018	0.226	−0.033	0.053
	Fat and Oil	0.034	0.079	0.006	0.783	0.028	0.083	−0.002	0.917
	Legumes	−0.041	0.024	−0.031	0.100	0.018	0.234	0.020	0.193
	Fruit	0.016	0.398	−0.001	0.980	0.053	0.001	0.029	0.081
	Green and Yellow Vegetables	0.028	0.108	0.023	0.212	0.031	0.033	0.005	0.761
	Other Vegetables	−0.068	<0.001	−0.061	0.001	−0.018	0.245	−0.029	0.071
	Mushrooms	0.005	0.769	0.000	0.979	0.015	0.278	0.005	0.754
	Seaweed	−0.014	0.402	−0.011	0.536	0.010	0.473	0.008	0.581
	Fish and Shellfish	0.026	0.158	0.043	0.031	0.009	0.541	0.017	0.304
	Meat and Poultry	0.062	0.002	0.062	0.005	0.056	<0.001	0.046	0.010
	Eggs	0.023	0.192	0.005	0.769	0.033	0.029	0.013	0.397
	Milk and Dairy Products	0.089	<0.001	0.070	<0.001	0.079	<0.001	0.045	0.005

^a^Age, body mass index, total energy intake and intake of each food group were entered in the model.

^b^Age, body mass index, total energy intake and intake of all food groups were entered in the model.

^c^Standardized regression coefficient.

Firstly, the associations with serum total cholesterol concentration were examined when each food group intake was separately entered into multiple linear regression analysis (model 1). In the 1980 survey, a significant negative association was found for intakes of rice, potatoes and other vegetables in both sexes, for legumes and seaweed only in men and for fish only in women. A significant positive association was found for wheat, fat and oil, meat, eggs and milk in both sexes and for sugar, fruit and green and yellow vegetables only in women. In the 1990 survey, a significant negative association was found for rice in both sexes and for potatoes, sweets and snacks, legumes and other vegetables only in men. A significant positive association was found for wheat, meat and milk in both sexes, for nuts and seeds only in men and for fruit, green and yellow vegetables and eggs only in women.

Secondly, the associations with serum total cholesterol concentration were examined when all food groups intake was collectively entered into multiple linear regression analysis (model 2). In the 1980 survey, a significant negative association was found for rice and potatoes in both sexes and for legumes and other vegetables only in men. A significant positive association was found for meat, eggs and milk in both sexes and for sugar only in women. In the 1990 survey, a significant negative association was observed for potatoes, sweets and snacks and other vegetables in men and for rice only in women. A significant positive association was found for meat and milk in both sexes and for nuts and seeds and fish only in men.

Intake of legumes, which was shown to be negatively correlated to serum total cholesterol concentration only in men, was also analyzed to determine the association with serum total cholesterol concentration among women aged 55 years or older (2098 in the 1980 survey and 1950 in the 1990 survey). Intake of legumes was not significantly associated with serum total cholesterol concentration, with a small negative standardized regression coefficient in each survey (standardized regression coefficient (β) = −0.031, *P* = 0.170 in the 1980 survey; β = −0.006, *P* = 0.786 in the 1990 survey).

## DISCUSSION

Food products known to contribute substantially to higher serum total cholesterol concentration include meat, eggs, milk, other dairy products and butter.^8^ Similarly, in the present study, meat, milk and eggs were also significantly positively associated with serum total cholesterol concentration in the representative sample of the Japanese population for the years 1980 and 1990. When the results of the 1980 and 1990 surveys were compared, intakes of eggs and milk and serum total cholesterol concentration were higher in the 1990 survey than in the 1980 survey, but the standardized regression coefficients of these food products in the 1990 survey were smaller than those in the 1980 survey. On the other hand, habitual intake of fish is known to be a negative predictor of serum total cholesterol.^9^ However, in the present study, statistically significant negative association between fish intake and serum total cholesterol concentration was found only in women in the 1980 survey, and positive association was observed in men in the 1990 survey.

In Japan, several national health promotion measures have become active for decades. Opportunities for medical check-ups was increased under the Law of Health and Medical Services for the Aged enacted in 1983 and people have more conscious of serum total cholesterol concentration which was add to the test items of the medical check-ups. The government established “Dietary Guidelines for Health Promotion” in 1985,^10^ and recommends, for example, taking various types of food for balanced nutrition and not consuming excessive fat, considering the quantity and type of fat consumed. Also, nutritional education recommending, for example, those with high serum total cholesterol concentration to reduce egg intake has become more common. Annual report of National Nutrition Survey in Japan conducted in 1988^11^ alerted an increase of osteoporosis in the elderly in context of low intake of calcium and new government established dietary guideline^12^ recommends taking calcium-rich foods, such as milk, small fish and seaweeds, for preventing osteoporosis. It is unsure that these health promotion measures influence the effect of food intake on serum total cholesterol concentration, however, several lifestyle and socioeconomic factors might have contributed to the effect of food intake on serum total cholesterol, and even make a reverse causality in some situations.

In the present study, the intake of legumes was inversely associated with serum total cholesterol concentration in men, but not in women of any age group or in women aged 55 years or older. Rosell et al found a significant negative association between the intake of soybeans and serum total cholesterol concentration in women, with a particularly strong association observed in postmenopausal women.^13^ Although we performed analysis among women aged 55 years or older, many of whom should have undergone menopause, we were not able to perform analysis in a population consisting only of postmenopausal women due to lack of information in the datasets about menopause.

When food group intake was separately entered into multiple linear regression analysis in the present study, the intake of rice decreased and that of wheat increased serum total cholesterol concentration in both sexes in both surveys. On the other hand, when food group intakes were collectively entered into the analysis, the intake of rice also decreased serum total cholesterol concentration in all groups except for men in the 1990 survey; however, no positive association was found between wheat and serum total cholesterol concentration. This suggests that the effect of food patterns, such as Japanese-type or Western-type meals, is greater than the direct effect of individual food groups, such as rice and wheat. Indeed, in a previous study examining the association between food patterns and serum total cholesterol concentration in Japanese, in which food patterns were divided into meat-based, vegetable-based and Western-type meals, higher levels of serum total cholesterol were found to be associated with meat-based and Western-type meals than with vegetable-based meals.^14^

In the present study, a negative association was also observed between vegetable intake and serum total cholesterol concentration. This might have been due to an increase in the intake of dietary fiber from vegetables, although Brown et al have suggested that the direct effect of dietary fiber in decreasing serum total cholesterol concentration is not so significant and that a “healthy food choice pattern” as represented by choosing food containing a large amount of dietary fiber instead contributes to the maintenance of serum total cholesterol concentration at an appropriate level.^15^ Future studies need to be performed from multiple viewpoints to promote disease prevention through improved dietary habits. Such studies should focus on nutrients, food groups or food pattern including food choice, which is more closely related to daily dietary habits.

Using the integrated datasets of the NIPPON DATA 80/90 and the National Nutrition Surveys in Japan conducted in 1980 and 1990, the present study has revealed positive and negative associations between serum total cholesterol concentration and food group intake in a representative sample of the Japanese population. Several limitations in estimating sex- and age-specific food intake using data based on the intake of whole family units and a cross-sectional design should be taken into consideration.^7^ Nevertheless, the results provide some insights into the improvements in dietary habits that can be made for disease prevention in Japan.
